# Nitric Oxide Synthase: Non-Canonical Expression Patterns

**DOI:** 10.3389/fimmu.2014.00478

**Published:** 2014-10-09

**Authors:** Joshua T. Mattila, Anita C. Thomas

**Affiliations:** ^1^Department of Microbiology and Molecular Genetics, University of Pittsburgh, Pittsburgh, PA, USA; ^2^Bristol Heart Institute and Bristol CardioVascular, Bristol Royal Infirmary, University of Bristol, Bristol, UK

**Keywords:** NOS1, NOS2, NOS3, iNOS, eNOS, nNOS, nitric oxide, nitric oxide synthase

## Abstract

Science can move ahead by questioning established or canonical views and, so it may be with the enzymes, nitric oxide synthases (NOS). Nitric oxide (NO) is generated by NOS isoforms that are often described by their tissue-specific expression patterns. NOS1 (nNOS) is abundant in neural tissue, NOS2 is upregulated in activated macrophages and known as inducible NOS (iNOS), and NOS3 (eNOS) is abundant in endothelium where it regulates vascular tone. These isoforms are described as constitutive or inducible, but in this perspective we question the broad application of these labels. Are there instances where “constitutive” NOS (NOS1 and NOS3) are inducibly expressed; conversely, are there instances where NOS2 is constitutively expressed? NOS1 and NOS3 inducibility may be linked to post-translational regulation, making their actual patterns activity much more difficult to detect. Constitutive NOS2 expression has been observed in several tissues, especially the human pulmonary epithelium where it may regulate airway tone. These data suggest that expression of the three NOS enzymes may include non-established patterns. Such information should be useful in designing strategies to modulate these important enzymes in different disease states.

## Introduction

Nitric oxide synthases (NOS) are enzymes that catalyze the conversion of l-arginine to l-citrulline and nitric oxide (NO), a free radical involved in homeostatic and immunological functions. There are three NOS isoforms and each isoform is associated with a set of characteristics and expression pattern. These expression patterns have been used to define the isoform’s nomenclature. NOS1 is often called nNOS because of its expression in neurons and the brain. NOS2 is referred to as iNOS, because its expression can be induced by cellular activation. NOS3 is often referred to as eNOS because of its association with the endothelium. The purpose of this Perspective is to examine the concept of inducible and constitutive NOS expression, and suggest that although the current paradigm is supported in many instances, the constitutive versus inducible dichotomy has been applied too broadly and may restrict our understanding of these enzymes’ functions in health and disease. A complete examination of potential NO-mediated physiological functions and NOS-expressing cells throughout an organism’s tissues is beyond the scope of this work. Our focus will be on immunologically relevant cells (e.g., lymphocytes, macrophages, and the epithelium), but we will also include some non-typical NOS-expressing cells (osteoclasts and cancer). Moreover, to avoid any confusion associated with the tissue-origin nomenclature, we will identify each NOS isoform by its numeric descriptor (e.g., NOS1, NOS2, NOS3).

## Basic NOS Biochemistry

All three NOS enzymes are catalytically active when dimerized and require two substrates, l-arginine, molecular oxygen, in combination with several co-factors including nicotinamide-adenine- dinucleotide phosphate (NADPH), flavin adenine dinucleotide (FAD), flavin mononucleotide (FMN), and (6R)5,6,7,8-tetrahydro-l-biopterin (BH4) to generate NO (1). Two NOS isoforms, NOS1 and NOS3, are commonly associated with constitutive expression. NOS1 and NOS3 activity is calcium dependent and requires interaction between the NOS enzyme and calmodulin-bound calcium to facilitate the catalysis of l-arginine and production of NO. In addition to the required co-factors and enzyme substrates, NOS1 and NOS3 are regulated through a variety of post-translational mechanisms including phosphorylation, myristoylation, and palmitoylation, and modification of subcellular localization (2, 3). NOS1 and NOS3 are commonly associated with the “low” levels of NO production that mediate intracellular signaling processes (NOS1) and vascular homeostasis (NOS3). In addition to NO production, NOS3 can function in an “uncoupled” manner and produce ROS when the available stores of BH4 are removed or oxidized, l-arginine depleted, or the NOS3 inhibitor asymmetric dimethyl-l-arginine overexpressed (1, 4). NOS1 and NOS3 are most commonly found in non-immunological cells (e.g., neurons, muscle, endothelium), and, because their NO output is relatively low, these isoforms are considered to be less immunologically important than their inducible, immunologically relevant counterpart, NOS2.

Inducible expression of NOS has long been associated with immunological functions. Immune cells use NO, often in conjunction with reactive oxygen intermediates (ROI), to kill pathogens and cancer cells (5, 6). NO acts non-specifically on a variety of targets and can kill targets at micromolar concentrations (7). This lack of specificity can cause collateral damage to normal cells and tissues and consequently, NO production is tightly regulated. NOS2 is minimally expressed or is not abundant intracellularly in macrophages unless immune-related stimulation and gene transcription occurs (hence its label as the “inducible” NOS isoform). Once transcribed, NOS2 has a high-affinity binding site for calmodulin and can function in a calcium-independent manner suggesting that any time it is expressed it is likely to be active. While the factors inducing and regulating NOS2 have been extensively studied in rodent models, NOS2 has been more difficult to study in primates. There has been controversy regarding its importance in human immune responses (8), or even whether NOS2 is expressed in human macrophages (9–11). There are several reasons why NOS2 expression has been difficult to identify in primate macrophages, including the different signals required for induction, inappropriate culture conditions, or intrinsic differences in NOS expression, but it is increasingly clear that NOS2 is expressed by human macrophages and has implications for human disease (12). A variety of immune cells other than macrophages [ranging from memory T cells (13, 14) to chondrocytes (15)] also express NOS2 in response to stimulation, suggesting that NOS2 expression is more flexible and extensive than previously reported.

## Is NOS Expression “True to Form?”

While NOS1, NOS2, and NOS3 have been associated with particular cell types and expression patterns, questions remain about whether these associations have been applied too strictly. Although the concept of constitutive (NOS1, NOS3) vs. inducible (NOS2) expression appears to be convenient, is it biologically plausible that NOS1 or NOS3 expression can be inducible under some circumstances, and conversely, can NOS2 be constitutively expressed in other circumstances? This issue has important clinical and therapeutic implications that need to be considered when designing new immunomodulatory therapies that rely on NOS expression to fight cancer or infectious diseases, or exploring current therapies for unanticipated effects. The answers are complicated by inconsistent data from experiments using different cell lines, animal models or clinical samples, and experimental techniques, but there are likely to be some generally applicable concepts and examples that we can use as guidelines. The remainder of this review will be focused on identifying the evidence for inducible NOS1 and NOS3, and constitutive NOS2 expression (summarized in Table 1).

**Table 1:** **Examples of non-canonical NOS expression in non-cancerous cells and tissues**.

Isoform	Cell type and reference	Species	Pathology	Expression pattern
NOS1	Bronchial epithelial cells (16)	Human	No	Ca^2+^ flux-dependent induction
NOS1	Alveolar macrophages (17)	Human	Tuberculosis	Induced-immune stimulation?
NOS1	Epithelioid macrophages (17)	human	Tuberculosis	Induced-immune stimulation?
NOS1	BMD^a^ macrophage (18)	mouse	N/A^b^	Ca^2+^ flux-dependent induction
NOS2	Colonic epithelium (19, 20)	Human	No	Constitutive expression
NOS2	Lung epithelium (21–23)	Human, macaque	No	Constitutive expression
NOS2	Brain, spinal tissue (24, 25)	Rat	No	Constitutive expression
NOS3	Alveolar macrophages (17, 26)	Human, macaque	Tuberculosis	Induced-immune stimulation?
NOS3	Epithelioid macrophages (17)	Human, macaque	Tuberculosis	Induced-immune stimulation?
NOS3	RAW264.7 macrophages^c^ (27)	Mouse	N/A	Ca^2+^ flux-dependent induction
NOS3	BMD^a^ macrophages (28)	Mouse	N/A	LPS-stimulated activity
NOS3	Osteoclasts (29)	human	No	Ca^2+^ flux-dependent induction

*^a^BMD, macrophages differentiated from bone marrow-derived monocytes*.

*^b^N/A, not applicable*.

*^c^RAW264.7 macrophages are a murine macrophage-like cell line*.

## Inducible Expression of “Constitutive” NOS Isoforms

There are a few instances of NOS1 expression that are clearly associated with upregulation in response to external stimuli. Classically, homeostatic NOS1 expression has been associated with neuronal signaling, although inflammatory stimuli can increase neuronal NOS1 expression, potentially leading to NO-mediated damage (30, 31). The relative contribution of NOS1 to pathology in this context is often confounded by co-induction of NOS2 expression. NOS1 splice variants are expressed in skeletal, cardiac, and smooth muscle cells and can generate NO that increases blood vessel dilatation and improved blood flow to nearby muscle tissue (32). The paucity of data on NOS1 expression in monocyte-derived macrophages has suggested, perhaps incorrectly, that NOS1 has little expression or importance for tissue macrophages. That said, NOS1 expression has been identified in human bronchoalveolar lavage cells (16), lung cancer (33), and alveolar and epithelioid macrophages from humans with tuberculosis (17). Although these observations do not necessarily indicate that NOS1 is upregulated in these cells, they demonstrate non-canonical NOS1 expression, and suggest that NOS1 may be immunologically important in unanticipated ways. Significantly, recent data indicate that NOS1 activity may be regulated post-transcriptionally, with important consequences for macrophage activation and function. In unprimed murine bone marrow-derived macrophages, immune complexes can stimulate calcium-dependent NOS1 and NOS3 activity that leads to increased phagocytosis by these cells (18) indicating the upregulated activity of the “constitutive” NOS isoforms that may have unappreciated roles in immunity. There may be other systems and cell types where post-transcriptional regulation of NOS1 expression through Ca^2+^-dependent or other modulatory mechanisms can confer inducible-like characteristics to this “constitutively” expressed isoform. However, identifying these mechanisms will require a deeper understanding of cellular dynamics and responses *in vivo*, and this cannot be obtained using immunohistochemistry or studying isolated cells or cell lines.

There is considerable evidence indicating NOS3 expression is inducible under the right conditions. Forstermann et al. found that expression of NOS3 could be modulated by a range of stimuli, and that there appeared to be a species-specific difference in NOS3 regulation (34). More recently, reports have identified that NOS3 expression can be induced in human and macaque macrophages (17, 26), but the significance of the presence and inducibility of this isoform in macrophages remains to be elucidated. The macrophage-like murine cell line RAW264.7 is known for its ability to produce significant quantities of NO via an iNOS-dependent mechanism following interferon gamma and LPS stimulation, but it also constitutively expresses calcium-sensitive NOS3 and produces low levels of NO in a calcium-dependent manner (27), reminiscent of NOS1-mediated NO production (18). The quantity of NO produced at steady state was approximately 20-fold less than that produced by NOS2 following stimulation, suggesting that its function was not directly bactericidal. A later study using murine bone marrow-derived macrophages identified NOS3-generated NO as an important factor in capacitating macrophage activation by enabling increased NK-κB activity, NOS2 expression, and NO production (28). Interestingly, mice lacking NOS3 produced less NOS2 protein and, subsequently, less NO following immune stimulation than control mice (28). In addition, it was observed that NOS2 induction led to diminished NOS3 expression, suggesting that there was an inverse feedback loop regulating NOS2- and NOS3-mediated NO production. NOS3 expression has also been observed in macrophages from non-human primates (26) and humans (17, 26) in the context of *Mycobacterium tuberculosis* infection, suggesting that NOS3 may be important in primate pulmonary immune responses. As previously mentioned, dysregulated NOS3 can produce ROS instead of NO and it cannot be ruled out that macrophage NOS3 does not generate non-traditional products instead of NO in these situations, particularly in an environments rich in l-arginine-utilizing enzymes (e.g., lung and tuberculous granuloma (26, 35)). There is also evidence that NOS3 may be important in bone remodeling and can be regulated by controlling access to Ca^2+^- and NOS2-mediated NO production (29). Unstimulated osteoclasts (macrophage-like cells responsible for bone remodeling) constitutively express both NOS2 and NOS3, with bone resorption associated with Ca^2+^-dependent NOS3-mediated NO production and inhibition of osteoclast function mediated by NOS2 (36). As with NOS1, it may be difficult to identify upregulated NOS3-mediated NO production in instances where this increase is attributable to post-translational events. There is also some evidence that NOS1 and NOS3 activity can be upregulated post-translationally by stimuli-specific release of Ca^2+^. This type of activation has important consequences in the regulation of many physiological processes, ranging from macrophage activation to bone homeostasis.

## Constitutive Expression of the “Inducible” NOS2 Isoform

**Figure 1:**
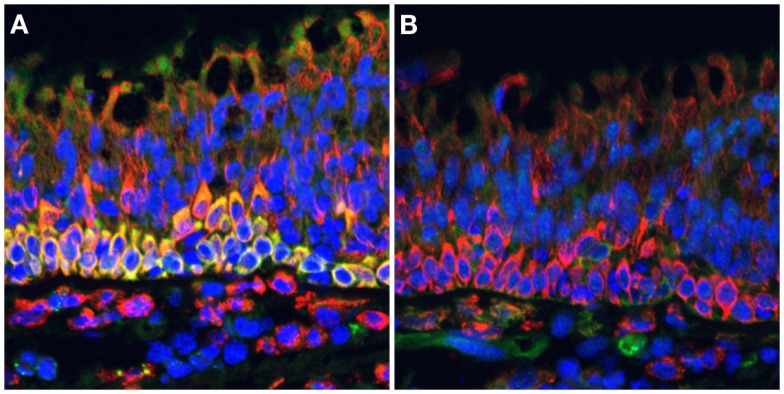
**NOS2 is strongly expressed by ciliated pseudostratified columnar epithelial cells in the cynomolgus macaque lung**. Formalin-fixed paraffin-embedded lung tissue sections were stained for **(A)** NOS2 (green) or **(B)** NOS3 (green) in combination with CD163 (red), a hemoglobin scavenger receptor expressed on macrophages and epithelial cells, and imaged by widefield epifluorescence microscopy. Intense NOS2 expression can be observed in the basal cells underlying the ciliated cells, with less intense staining in the ciliated cells. NOS3 staining is associated with cells in the lamina propria but not ciliated epithelial cells. This staining is characteristic of ciliated epithelia of both uninfected and *Mycobacterium tuberculosis*-infected macaques (pictured). DAPI-stained nuclei are indicated in blue.

Nitric oxide synthase 2 has become the paradigm of an inducible immunoresponsive gene, particularly in rodent systems. The high-affinity calmodulin-binding domain of NOS2 enables it to function in conditions where Ca^2+^ is unavailable, suggesting that dimerized NOS2 is always active and capable of generating NO when the appropriate co-factors are present (1, 37). The ease at which NOS2 expression is induced varies across and there are significant differences in species-specific expression patterns (9, 38, 39) and even differences between individuals in genetically diverse populations (40). In mice, which are often viewed as the paradigm for inducible NOS2 expression, some strains have macrophages that readily express NOS2 when stimulated, whereas other strains have more restrained NOS2 expression (41). NOS2 expression in primate systems appears to have different requirements for its induction that can result in NO concentrations that differ by several orders of magnitude (39). That said, although NOS2 expression is generally inducible, in some circumstances, NOS2 can be constitutively expressed. Some of the best-described examples of constitutive NOS2 expression occur in the human colonic epithelium (19, 20) and pseudostratified columnar epithelia in the human (21–23) and non-human primate lung (Figure 1). In the lung, constitutive NOS2 expression by these cells is robust and likely to be responsible for the majority of exhaled NO in human breath (22). NOS2 expression from these cells is thought to help regulate ciliary beat (16) and airway tone or reactivity (22). Rat epithelium can also express NOS1 (21), suggesting that there are likely to be species-specific differences in epithelial NOS expression. It should also be noted that neither the lung nor the colonic epithelia are sterile environments, and there remains the possibility that NOS2 expression occurs in response to stimulation by the normal microbiota associated with these tissues. Neural tissue is much less likely to be associated with bacteria, and there is evidence that NOS2 in rodents is constitutively expressed at low levels in brain and spinal tissue (24, 25). This can be upregulated above basal levels by inflammatory stimuli where it may be associated with disease in models of pathological conditions including Alzheimer’s disease and arthritis-associated arthralgia (30, 31).

In addition to constitutive expression in normal tissues, constitutive NOS2 expression has been identified in tumors, including melanoma (42), prostate cancer (43), colorectal cancer (44), breast cancer (45), bladder cancer (46), head and neck cancer (47), and esophageal adenocarcinoma (48). In these pathologies, NOS2 is often associated with poor prognosis, potentially related to increased angiogenesis, metastatic ability, aggressive growth, resistance to apoptosis, and chemotherapy (49, 50). The mechanistic basis for why tumor progression is sometimes associated with NOS2 expression is not fully understood, but could include additional mutation by NOS-mediated DNA strand breakage, and immunosuppression of T-cell responses through both NO-dependent and NO-independent mechanisms (49). Research in this area is not without controversy and there is evidence that NOS2-generated NO has protective effects in cancer, possibly reflecting differences in a tumor’s inflammatory state, the type of infiltrating immune cells, tumor location, tumor type, and the stage of disease, as well as differences in whether there are high or low levels of NO in the tumor microenvironment (49, 50). Although poorly understood at present, a better understanding of how NOS2 expression influences the tumor environment may lead to the development of novel interventional strategies and improved clinical treatment (50, 51).

## Concluding Statement

A better understanding of the properties and expression patterns of the different NOS isoforms has shed light on the diverse range of physiological roles that these enzymes fulfill. We now know that there are instances where functions of these enzymes diverge from the dichotomous constitutive or inducible expression patterns they are often associated with. We should take this opportunity to study the full range of possible NOS function. Recognizing the possibility that NOS enzymes may act in non-canonical ways can only increase our understanding of how tissues respond to disease and give us new opportunities for developing innovative therapeutic strategies.

## Author Contributions

Joshua T. Mattila and Anita C. Thomas contributed to drafting the manuscript.

## Conflict of Interest Statement

The authors declare that the research was conducted in the absence of any commercial or financial relationships that could be construed as a potential conflict of interest.
